# *Bifidobacterium breve* UCC2003 metabolises the human milk oligosaccharides lacto-N-tetraose and lacto-N-neo-tetraose through overlapping, yet distinct pathways

**DOI:** 10.1038/srep38560

**Published:** 2016-12-08

**Authors:** Kieran James, Mary O’Connell Motherway, Francesca Bottacini, Douwe van Sinderen

**Affiliations:** 1 School of Microbiology, University College Cork, Cork, Ireland; 2 APC Microbiome Institute, University College Cork, Cork, Ireland

## Abstract

In this study, we demonstrate that the prototype *B. breve* strain UCC2003 possesses specific metabolic pathways for the utilisation of lacto-N-tetraose (LNT) and lacto-N-neotetraose (LNnT), which represent the central moieties of Type I and Type II human milk oligosaccharides (HMOs), respectively. Using a combination of experimental approaches, the enzymatic machinery involved in the metabolism of LNT and LNnT was identified and characterised. Homologs of the key genetic loci involved in the utilisation of these HMO substrates were identified in *B. breve, B. bifidum, B. longum* subsp. *infantis* and *B. longum* subsp. *longum* using bioinformatic analyses, and were shown to be variably present among other members of the *Bifidobacterium* genus, with a distinct pattern of conservation among human-associated bifidobacterial species.

Consumption of maternal breast milk, or the lack thereof, influences the gut microbiota composition of the neonate^1^,^2^,^3^. Incorrect development or disruption of this microbial community contributes to disorders such as Necrotising Enterocolitis, infantile diarrhoea and Group B streptococcal neonatal infection^4^,^5^,^6^,^7^,^8^. Strikingly, the faecal microbiota of healthy breastfed infants is enriched for certain species of the *Bifidobacterium* genus^9^, which are high-G+C Gram-positive anaerobes and members of the *Actinobacteria* phylum. Naturally found as symbionts of the mammalian, avian or insect digestive tract, bifidobacteria enjoy substantial scientific attention due to their purported beneficial properties^2^,^10^,^11^,^12^,^13^,^14^,^15^,^16^.

While lactose (Galβ1-4Glc) comprises the main carbohydrate component of human breast milk and colostrum (~90%), human milk oligosaccharides (HMOs) constitute the next most significant carbohydrate fraction, ahead of glycolipids^2^,^17^, and are typically found at a concentration of ≥4 g/L (and as high as 15 g/L)^2^,^18^,^19^,^20^. HMOs represent a heterogeneous glycan mix, of which >200 distinct structures have been identified^18^. The majority of these HMO structures are classified into 2 types. The abundant Type I HMOs contain lacto-N-tetraose (LNT; Fig. 1) (Galβ1-3GlcNAcβ1-3Galβ1-4Glc), which is composed of a lactose coupled to lacto-N-biose (LNB) (Galβ1-3GlcNAc). Type II HMOs contain the LNT isomer lacto-N-neotetraose (LNnT; Fig. 1) (Galβ1-4GlcNAcβ1-3Galβ1-4Glc), which is composed of lactose linked to N-acetyllactosamine (LacNAc) (Galβ1-4GlcNAc), an isomer of LNB. Larger Type I and II HMOs may contain further LNB or LacNAc subunits, and can be fucosylated or sialylated^2^,^18^,^21^.

Despite the abundance of HMOs in breast milk, these glycans cannot be metabolised by the infant, and it is currently believed that they facilitate the establishment of an infant-specific gut microbiota, with bifidobacteria being particularly abundant^16^,^22^. Common among the latter are *Bifidobacterium bifidum, Bifidobacterium longum* subsp. *infantis* and subsp. *longum, Bifidobacterium breve, Bifidobacterium pseudocatenulatum* and *Bifidobacterium kashiwanohense*^9^,^23^,^24^,^25^,^26^,^27^. Unsurprisingly, it has been shown that certain bifidobacterial species can metabolize (particular) HMOs^18^,^28^,^29^,^30^,^31^,^32^,^33^,^34^. Previous studies have elucidated some of the metabolic pathways for HMO utilisation by *B. bifidum* and *B. longum* subsp. *infantis*, with particular focus on LNT and LNnT^28^. *B. longum* subsp. *infantis* internalises particular, intact small-mass HMOs, including (precursors of) LN(n)T^18^,^28^,^35^, which are in turn hydrolysed into lacto-N-triose and galactose, by two HMO type-specific β-galactosidases (i.e. one enzyme acting on LNT, the other on LNnT)^29^. Lacto-N-triose is further hydrolysed by an N-acetylhexosaminidase into N-acetylglucosamine (GlcNAc) and lactose, the latter of which is then hydrolysed by a β-galactosidase^30^ into galactose and glucose, to enter the Leloir and fructose-6-phosphate (F6P) phosphoketolase pathways and amino-sugar metabolising pathway (for GlcNAc)^28^,^31^, all of which feed into the overall *Bifidobacteriaceae*-specific metabolic pathway known as the Bifid Shunt.

*B. bifidum* possesses two distinct, and apparently unique pathways for the metabolism of LN(n)T. Large type I and II HMOs are degraded by extracellular fucosidases, sialidases and glycoside hydrolases to release LNT and LNnT^28^. LNT is hydrolysed at its central β-1,3-link by an extracellular glycoside hydrolase into lactose and LNB, the latter being transported into the cell and then degraded by two distinct LNB phosphorylases (LNBP), releasing galactose 1-phosphate and GlcNAc^32^,^33^. The released lactose is either hydrolysed by an extracellular β-galactosidase into galactose and glucose (which are both internalized by the cell), or transported into the cell, where it is similarly hydrolysed by intracellular β-galactosidases^28^,^32^,^36^. These monosaccharides are then further metabolised by the same pathways as those described for *B. longum* subsp. *infantis*. This type I HMO metabolism has also been observed in some species of *B. longum* subsp. *longum*^32^,^37^. In addition, *B. bifidum* possesses a separate pathway to degrade and utilise LNnT. An extracellular β-galactosidase cleaves LNnT at its Galβ-1,4 residue, liberating galactose and lacto-N-triose^34^. The lacto-N-triose is then further hydrolysed by an extracellular N-acetylhexosaminidase, releasing GlcNAc and lactose, with the latter further hydrolysed by the aforementioned extracellular β-galactosidases into glucose and galactose (which are transported into the cell)^36^, or internalised and then degraded as described. Once within the cell, these monosaccharides are metabolised as mentioned above^34^.

It should be noted that a specific pathway exists for LNB metabolism, known as the GNB/LNB pathway. In *B. bifidum*, as mentioned above, LNB is phosphorolysed into monosaccharides by either of two different LNBP enzymes, while in *B. infantis*, LNB is phosphorolysed by a single LNBP enzyme, whose gene shares homology with both *B. bifidum* LNBP genes^28^,^31^. This GNB/LNB pathway appears to be present in bifidobacterial species commonly found in infant faeces^28^. The apparent absence of this GNB/LNB pathway and, specifically, the LNBP-encoding gene in adult-associated bifidobacteria (such as *B. adolescentis*) is manifested through their inability to utilise LNB or other HMOs as a carbon source for growth, and may therefore explain, at least in part, their absence or low abundance in the microbiota of breast-fed infants^18^.

Little information exists regarding HMO utilisation by *B. breve*, although it has been suggested that *B. breve* acts as a ‘scavenger’ through cross-feeding on HMO-derived monosaccharides that are released due to the extracellular hydrolytic activities produced by other infant gut microbiota members^18^. However, more recent studies have suggested that *B. breve* is able to utilise particular HMOs, such as fucosyllactose, LNT and sialyl-LNT, or derived structures such as LNB and sialic acid^17^,^38^,^39^,^40^.

In this study, we show that *B. breve* possesses the metabolic machinery for the degradation and utilisation of LNT and LNnT. Furthermore, we assess the presence and distribution of key gene loci involved in LNT and LNnT utilisation across members of the *Bifidobacterium* genus.

## Results

### Growth of *B. breve* strains on LNT and LNnT

In order to determine if *B. breve* strains are capable of LNT and/or LNnT metabolism, growth in modified MRS medium (mMRS) supplemented with either 1% (wt/vol) LNT, LNnT or lactose (as a positive control) was assessed for sixteen *B. breve* strains by measuring the OD_600nm_ following 24 hours of anaerobic growth at 37 °C. All tested *B. breve* strains were generally observed to grow well (final OD_600nm_ > 0.8) on both LNT and LNnT, with some variability between strains on one or both HMO substrates (Supplemental Fig. S1).

### Transcriptome analysis of *B. breve* UCC2003 grown on LNT and LNB

In order to identify genes that are involved in the metabolism of the Type I HMO central moiety LNT and its constituent component LNB, global gene expression was determined by microarray analysis during growth of *B. breve* UCC2003 in mMRS supplemented with LNT or LNB, and compared to the transcriptome of the strain when grown in mMRS supplemented with ribose. Ribose was selected as a suitable transcriptomic reference, as the metabolic pathway and gene expression profile for growth of UCC2003 on ribose is known and has been employed previously as a reference^39^,^41^. Genes that were shown to be significantly upregulated in transcription above the designated cut-off (fold-change >2.5, P < 0.001) are shown in Table 1. The genes upregulated in expression included those corresponding to the loci Bbr_0526-530, Bbr_1551-1553, Bbr_1554-1560 and Bbr_1585-1590. The possible involvement of these genes in LNT/LNB metabolism will be further discussed below.

### Genetic organisation of the genes involved in metabolism of LNT

**Figure 2:**
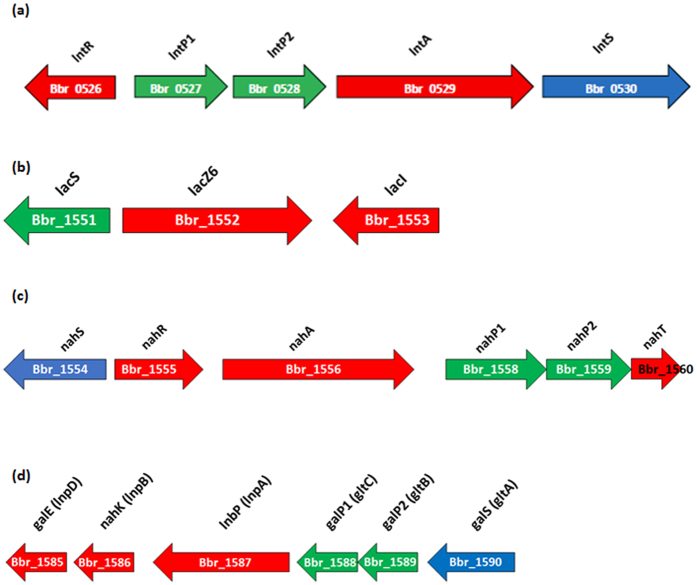
Schematic representation of the gene loci involved in the utilisation of LNT, LNnT and their substituents in *B. breve* UCC2003, as based on transcriptome analysis. The length of the arrows is proportional to the size of the open reading frame and the gene locus name, which is indicative of its putative function, is given at the top. Genes shown in red are predicted to encode proteins with an intracellular localisation, genes shown in green are predicted to encode proteins with a transmembrane localisation, and genes shown in blue are predicted to encode proteins with an extracellular localisation and a signal peptide sequence.

Based on the microarray results and functional prediction of these LNT (and LNB)-upregulated genes, we implicate the gene clusters Bbr_0526-0530, Bbr_1554-1560, Bbr_1585-1590 and possibly Bbr_1551-1553 (outlined in Fig. 2) in LNT and LNB metabolism in *B. breve* UCC2003.

Bbr_0527 and Bbr_0528 (designated here as *lntP1* and *lntP2*, respectively) are both predicted to encode permease components of an ABC transporter system. Also located in this cluster (Fig. 2) is Bbr_0529 (designated *lntA*), which encodes a predicted β-galactosidase of the GH42 glycoside hydrolase family. Bbr_0530 (designated here as *lntS*) encodes a putative solute-binding protein of an ABC transporter system. Located immediately upstream of this cluster, Bbr_0526 (designated *lntR*) encodes a putative LacI-type transcriptional regulator. We have previously implicated the Bbr_0526-530 gene cluster, in the metabolism of galacto-oligosaccharides^42^, where the genes were designated *gosR (lntR*), *gosD (lntP1*), *gosE (lntP2*), *gosG (lntA*) and *gosC (lntS*). Here, we chose to re-designate this cluster as the *lnt* cluster, as its primary function appears to be in LNT and LNnT metabolism (see below).

Bbr_1551 (designated here as *lacS*) encodes a galactoside symporter, and is predicted to function in the transport of lactose and galacto-oligosaccharides into the cell. The *lacS* gene is located in a cluster that also contains genes Bbr_1552 (designated *lacZ6*), a β-galactosidase (GH2) previously shown to be involved in galacto-oligosaccharide metabolism^42^, and Bbr_1553 (designated *lacI*), a lacI-type regulator.

Bbr_1555 (designated *nahR*) is predicted to encode a Nag-type transcriptional regulator. Bbr_1556 (designated *nahA*) encodes a putative β-N-acetylhexosaminidase (GH20). Upstream of *nahR* is a gene encoding a putative solute binding protein (Bbr_1554 and designated here as *nahS*), while located downstream of *nahA* are Bbr_1558 (*nahP1*), Bbr_1559 (*nahP2*) and Bbr_1560 (*nahT*), which are predicted to specify two permeases and an ATP-binding protein; respectively (Fig. 2).

Bbr_1587 (designated here as *lnbP*) encodes a clear homolog (89.84% similarity to BBPR_1055 of *B. bifidum* PRL2010, and 97.62% to Blon_2174 of *B. longum* subsp. *infantis* ATCC 15697) of the previously characterised LNBP, which belongs to the 1,3-β-Galactosyl-N-acetylhexosamine phosphorylase family (GH112)^31^,^43^,^44^. The presumed function of this protein in UCC2003 is the cleavage and concomitant phosphorylation of LNB, and its passage into the GNB/LNB pathway^43^,^44^,^45^,^46^,^47^. The *lnbP* gene is located in the cluster Bbr_1585-1590, which also includes genes encoding a UDP-glucose 4-epimerase (Bbr_1585; *galE*), a phosphotransferase family protein (Bbr_1586; *nahK*), two permease proteins (Bbr_1588 and Bbr_1589; *galP1* and *galP2*, respectively), and a solute-binding protein (Bbr_1590; *galS*) (Fig. 2), which are all predicted to function in the metabolism of GNB/LNB.

### Heterologous expression, purification and biochemical characterisation of LntA and NahA, and enzymatic activity on LNT

**Figure 3:**
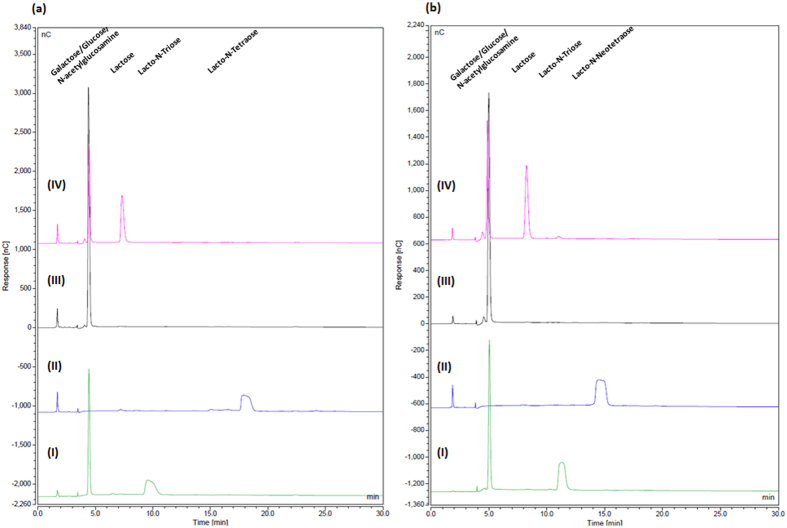
HPAEC chromatogram profiles of (**a**) LNT and (**b**) LNnT, when incubated in MOPS buffer (pH7) with: (I) LntA alone, (II) NahA alone, (III) LntA and NahA together, and (IV) LntA, followed by a denaturation step and the subsequent addition of NahA.

In order to investigate the predicted enzymatic activities encoded by *lntA* (Bbr_0529) and *nahA* (Bbr_1556) on core Type I HMO structure LNT, the corresponding LntA and NahA proteins were purified as His-tagged versions (LntA_His_ and NahA_His_; see Materials and Methods). Biochemical and substrate specificity characterisations were performed by incubating LntA_His_ and NahA_His_ on their own or in combination with LNT, and analysing the reaction products by HPAEC-PAD. Purified LntA_His_ was shown to remove the galactose moiety at the non-reducing end of the substrate LNT (Fig. 3a), demonstrating hydrolytic activity towards Galβ-1,3GlcNAc in Type I HMO structures, and indicating a key role in the hydrolysis and utilisation of LNT.

When NahA_His_ was incubated alone with LNT, no degradation of the tetrasaccharide structure was observed (Fig. 3a). However, when LntA_His_, and NahA_His_ were together incubated with LNT, complete breakdown of LNT to the monosaccharide constituents was observed. When LNT was incubated first with LntA_His_, followed by an enzymatic heat denaturation step, and then incubated with NahA_His_, different reaction product profiles were observed. Samples taken following the initial denaturation prior to the addition of NahA_His_ showed the presence of lacto-N-triose and galactose. Samples then taken following subsequent incubation with NahA_His_ indicated the presence of galactose, GlcNAc and lactose.

These results show that LntA hydrolyses LNT, releasing galactose and lacto-N-triose. Lacto-N-triose is then hydrolysed by NahA, liberating Lactose and GlcNAc. The lactose is then further broken down by LntA (and probably other β-Galactosidases *in vivo*), releasing galactose and glucose.

### Phenotypic analysis of *B. breve* strains harbouring mutations of genes implicated in LNT metabolism

**Figure 4:**
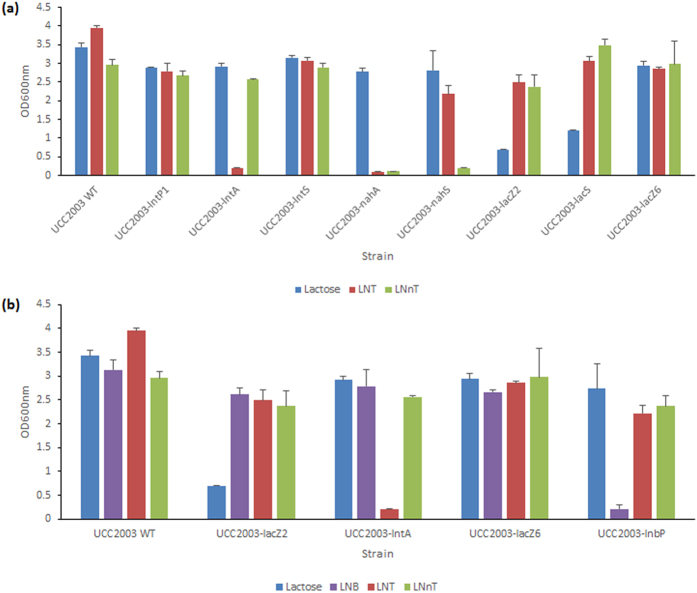
(**a**) Final OD_600nm_ values after 24 hours of growth of wild type *B. breve* UCC2003 and mutants *B. breve* UCC2003-lntP1, *B. breve* UCC2003-lntA, *B. breve* UCC2003-lntS, *B. breve* UCC2003-nahA, *B. breve* UCC2003-nahS, *B. breve* UCC2003-lacZ2, *B. breve* UCC2003-lacS and *B. breve* UCC2003-lacZ6 in modified MRS containing 1% (wt/vol) lactose, 1% (wt/vol) LNT or 1% (wt/vol) LNnT as the sole carbon source. (**b**) Final OD_600nm_ values after 24 hours of growth of wild type *B. breve* UCC2003, and mutants *B. breve* UCC2003-lacZ2, *B. breve* UCC2003-lntA, *B. breve* UCC2003-lacZ6, and *B. breve* UCC2003-lnbP in modified MRS containing 1% (wt/vol) lactose, 1% LNB, 1% (wt/vol) LNT, 1% (wt/vol) LNnT as the sole carbon source. The results are the mean values obtained manually from two separate experiments (due to the limited availability of certain carbohydrates). Error bars represent the standard deviation.

In order to investigate if disruption of individual genes of the *lnt* cluster affect the ability of UCC2003 to utilise LNT, a number of insertion mutants, which either had been generated previously, or which were constructed here, were assessed. An insertion mutant was constructed in *lntS*, resulting in strain *B. breve* UCC2003-lntS. Insertion mutants in *lntP1* and *lntA*, generating strains *B. breve* UCC2003-lntP1 and *B. breve* UCC2003-lntA, respectively, had been generated in a previous study (then designated *B. breve* UCC2003-gosD and *B. breve* UCC2003-gosG, respectively)^42^. These strains were analysed for their ability to grow in mMRS supplemented with LNT or LNB, with lactose controls, as compared to *B. breve* UCC2003. A complete lack of growth was observed for *B. breve* UCC2003-lntA in media containing LNT, in contrast to normal growth by the wild type in the same media (Fig. 4a). Growth of this mutant strain was not impaired in media containing LNB (not shown). As expected, reintroduction of the *lntA* gene on plasmid pBC1.2 under the control of the constitutive p44 promoter^48^ (see Materials and Methods) in the UCC2003-lntA mutant restored the mutant’s ability to grow on LNT (Supplemental Fig. S3). Thus, transcriptome data, substrate hydrolysis profiles and mutant growth results demonstrate that this β-galactosidase is specifically required for the hydrolysis of the Type I HMO central moiety LNT at its Galβ1-3GlcNAc residue, liberating galactose and lacto-N-triose for further metabolic processing. Insertion mutants *B. breve* UCC2003-*lntP1* and *B. breve* UCC2003-*lntS* were shown to reach the same final optical density as wild type strain UCC2003 during growth in mMRS supplemented with LNT(Fig. 4a), indicating that either these predicted transport components do not play a role in LNT metabolism, or that there are compensatory transport systems for this substrate.

In order to investigate if disruption of *lnbP* affects the ability of UCC2003 to utilise LNT and/or LNB, a Tn5 transposon insertion mutant of *lnbP* (designated *B. breve* UCC2003-lnbP) was adopted from a previous study^46^ and compared to wild type *B. breve* UCC2003 for its ability to grow in mMRS broth supplemented with LNT or LNB, or lactose as control. As expected, and in contrast to the wild type control, *B. breve* UCC2003-*lnbP* displayed a near total inability to grow on LNB (Fig. 4b). This mutant reached final OD_600nm_ levels on LNT and lactose that are comparable to the wild type strain (Fig. 4b), confirming the crucial role of *lnbP* in LNB metabolism, while it also shows that *lntA* plays no direct *in vivo* role in LNB metabolism.

In order to investigate if disruption of *lacS, nahS* or *nahA* affects the ability of UCC2003 to utilise LNT, insertion mutants in these genes were assessed. The insertional mutant *B. breve* UCC2003-lacS^42^ did not show any significant difference in the final OD reached following growth on LNT as compared with the wild type (Fig. 4a). The insertional mutant in *nahS* (generated in this study), designated *B. breve* UCC2003-nahS, also did not exhibit a difference in final OD following growth in media containing LNT (as compared to the wild type, (Fig. 4a). This suggests that either *nahS* is not involved in LNT transport, or that while *nahS* and the other transport system components of the *nah* locus may be involved in the transport of LNT into the cell, their function is compensated by the activity of one or more other transport systems. The insertion mutant in *nahA* (generated in this study), designated *B. breve* UCC2003-nahA, was shown to exhibit a complete lack of growth in LNT-containing media (Fig. 4a). Reintroduction of the *nahA* gene *in trans* on plasmid pBC1.2, under the transcriptional control of its own promoter^48^ (see Materials and Methods), in the UCC2003-nahA mutant restored the ability to grow on LNT (Supplemental Fig. S3). These findings demonstrate that *nahA* is crucial for LNT metabolism, being responsible for the hydrolysis of lacto-N-triose, thereby liberating lactose and GlcNAc.

### Transcriptome analysis of *B. breve* UCC2003 grown on LNnT, lactosamine and lactose

In order to investigate which genes are involved in the metabolism of Type II central moiety LNnT and its constituent component LacNAc, global gene expression was determined by microarray analysis during growth of *B. breve* UCC2003 in mMRS supplemented with each respective sugar, as well as lactose (which also possesses a Galβ-1,4 residue) as compared with gene expression during growth in mMRS supplemented with ribose (NB. We used a hydrochloride salt of lactosamine instead of LacNAc, as the latter was not commercially available in an affordable quantity). Genes that were shown to be significantly upregulated in transcription above the designated cut-off (fold-change >2.5, P < 0.001) are shown in Table 1. The genes upregulated in expression included those located in the loci Bbr_0526-530, Bbr_1551-1553, Bbr_1554-1560 and Bbr_1585-1590. Possible involvement of these genes in LNnT/LacNAc metabolism are assessed below.

### Genetic organisation of the genes involved in metabolism of LNnT

Based on the results of the microarray analyses performed and functional annotation of LNnT/LacNAc-upregulated genes, we propose that the products of the gene clusters Bbr_0526-0530, Bbr_1551-1553 and Bbr_1554-1560 (schematically outlined in Fig. 2) are involved in the metabolism of LNnT and LacNAc (present as central moieties in Type II HMO) in *B. breve* UCC2003.

### Heterologous expression, purification and biochemical characterisation of LntA, LacZ2, LacZ6, NahA, and enzymatic activity on Type II HMO structure LNnT

In order to investigate the predicted individual and combined enzymatic activities of the protein products *lacZ2* (Bbr_0010), *lntA* (Bbr_0529), *lacZ6* (Bbr_1552) and *nahA* (Bbr_1556) on core Type II HMO structure LNnT, the corresponding His-tagged protein products were overproduced and purified. Biochemical and substrate specificity characterisations were performed by incubating individual enzymes or combinations thereof with a particular substrate, and analysing the reaction products by HPAEC against a number of substrate standards and reaction controls. Purified LntA_His_ was capable of removing the galactose moiety at the non-reducing end of LNnT (Fig. 3b), as well as lactose (data not shown), demonstrating a triple specificity for Galβ-1,3GlcNAc, Galβ-1,4GlcNAc and Galβ-1,4Glc glycosidic linkages, and thus both Type I and Type II HMO central moieties and lactose. Purified LacZ2_His_ and LacZ6_His_ were, under the conditions applied, also capable of hydrolysing LNnT and lactose (data not shown).

When both LntA_His_ and NahA_His_ were incubated with LNnT, complete hydrolysis of LNnT to its constituent monosaccharides was observed. When LNnT was incubated first with LntA_His_, followed by an enzymatic heat denaturation step, and then incubated with NahA_His_, different reaction product profiles were observed. Samples taken following the initial denaturation prior to the addition of NahA_His_ showed the presence of lacto-N-triose and galactose. Samples taken following the addition of NahA_His_ and subsequent incubation showed the presence of galactose, GlcNAc and lactose (Fig. 3b).

Similar results from separate and combined reactions were obtained using LacZ2_His_ or LacZ6_His_, together with NahA_His_ on the substrate LNnT (Supplemental Fig. S2).

These results agree with the model for Type II HMO metabolism proposed here; where LntA, LacZ2 and/or LacZ6 (and perhaps other β-galactosidases) hydrolyse LNnT, releasing galactose and lacto-N-triose, unlike LNT, which neither purified LacZ2 nor LacZ6 displayed the ability to hydrolyse (data not shown). Lacto-N-triose is then hydrolysed by NahA, liberating lactose and GlcNAc. Lactose is then further broken down by LntA (and other β-Galactosidases, including LacZ2 and LacZ6, *in vivo*), releasing galactose and glucose. Therefore, these two β-Galactosidases may carry out hydrolysis of Type II HMO central moieties (in conjunction with LntA).

### Phenotypic analysis of *B. breve* strains harbouring mutations of genes implicated in LNnT metabolism

In order to investigate if disruption of individual genes of the *lnt* cluster affects the ability of UCC2003 to utilise LNnT, a number of insertion mutants were assessed. *B. breve* UCC2003-lntP1, *B. breve* UCC2003-lntS and *B. breve* UCC2003-lntA were analysed for their ability to grow in mMRS supplemented with LNnT with lactose controls, as compared to wild type *B. breve* UCC2003. *B. breve* UCC2003-lntA reached the same final optical density following growth in media containing LNnT compared to wild type UCC2003 (Fig. 4a). Transcriptome data and carbohydrate hydrolysis assays (see above) demonstrated that *lntA* is involved in LNnT metabolism, but its function can also be carried out by other glycoside hydrolases, as mentioned. The insertion mutants *B. breve* UCC2003-lntP1 and *B. breve* UCC2003-lntS did not show any significant impairment in growth on either LNnT or lactose, as compared with the wild type (Fig. 4a), indicating that either these predicted transport components do not play a role in LN(n)T metabolism or that there are additional transport systems that allow internalisation LNnT. It is most likely that *lntP1, lntP2* and *lntS* do indeed also function in the transport of extracellular LNnT into the cytoplasm, but that their function can be supplemented or indeed supplanted by other cellular transport systems. The *lnbP* insertion mutant, *B. breve* UCC2003-*lnbP*, was shown to reach final OD_600nm_ levels on either lactose or LNnT that were comparable to those of the wild type strain (Fig. 4b), suggesting *lnbP* plays no direct role in LNnT utilisation.

In order to investigate if disruption of each of the five individual genes *lacZ2, lacZ6, lacS, nahS* and *nahA* affects the ability of UCC2003 to utilise LNnT, mutants of these genes were assessed. The *nahS* and *nahA* insertion mutants mentioned above were used for this purpose, in addition to insertion mutants in the genes Bbr_1551 (*lacS*), Bbr_1552 (*lacZ6*), constructed in a previous study^42^, as well as a Tn5 transposon mutant in Bbr_0010 (*lacZ2*), from a separate study^49^. These mutant strains were analysed for their ability to grow in mMRS supplemented with LNnT with a lactose (and ribose, in the case of *B. breve* UCC2003-lacZ2 and *B. breve* UCC2003-lacS) control, as compared to *B. breve* UCC2003. The insertion mutants *B. breve* UCC2003-lacZ2 and *B. breve* UCC2003-lacZ6 did not show any significant impairment in growth on LNnT, as compared to the wild type, as neither did the insertional mutant *B. breve* UCC2003-lacS (Fig. 4a). While these growth analyses indicate that *lacZ2* and *lacZ6* are not essential for LNnT metabolism, the hydrolysis assay results and LNnT-dependent transcriptional induction of *lacZ6* (described above) suggest that these two glycoside hydrolases play a role in LNnT hydrolysis, presumably in concert with *lntA*. The insertion mutant *B. breve* UCC2003-nahS was shown to exhibit a near total lack of growth in media supplemented with LNnT, as compared to the wild type grown in the same media (Fig. 4a). As expected, reintroduction of the *nahS* gene on plasmid pBC1.2, under the regulation of its own promoter^48^ (see Materials and Methods), in the UCC2003-nahS mutant reverted the mutant’s (near complete) inability to grow on LNnT (Supplemental Fig. S3). This indicates that *nahS* encodes the solute binding protein predominantly required for the uptake of LNnT, and that the *nah* locus-encoded transport system is of critical importance for the transport of the Type II central tetrasaccharide into the cell. In contrast to the wild type, *B. breve* UCC2003-nahA failed to grow in media containing LNnT (Fig. 4a). As expected, reintroduction of the *nahA* gene on plasmid pBC1.2, under the regulation of its own promoter^48^ (see Materials and Methods), in the UCC2003-nahA mutant reverted the mutant’s inability to grow on LNnT (Supplemental Fig. S3). This result demonstrates the essential role of the *nahA* product in LNnT metabolism, by hydrolysing lacto-N-triose at its GalNacβ1-3Gal linkage, liberating lactose and GlcNAc.

Growth of the insertion mutants was not impaired on lactose, where all strains reached final OD_600nm_ levels comparable to that reached by UCC2003, except for *B. breve* UCC2003-lacZ2 and *B. breve* UCC2003-lacS, as the interrupted genes in these mutants are known to be crucial for lactose metabolism^42^,^49^ (Fig. 4a). *B. breve* UCC2003-lacZ2 and *B. breve* UCC2003-lacS did reach final OD_600nm_ values similar to that of UCC2003 when grown on ribose (data not shown).

### Distribution of HMO central moiety utilisation-associated genes across the *Bifidobacterium* genus

**Figure 5:**
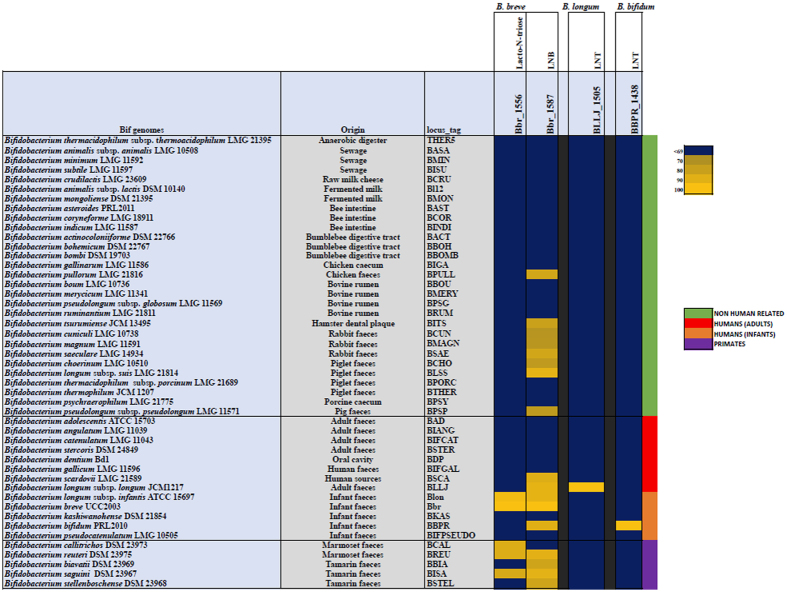
Heatmap representing the distribution of homologs of two genes from *B. breve* UCC2003, one gene from *B. longum* subsp. *longum* JCM1217 and one gene from *B. bifidum* PRL2010 across the *Bifidobacterium* genus. Gene products from the representative strain genomes of all online-available *Bifidobacterium* species with a significant homology of 70% iterative similarity over 50% of protein length are represented in the matrix, which employs a code colour grading that represents the degree of sequence similarity, with species ordered by origin of isolation. Bbr_1556 (*nahA*) and Bbr_1587 (*lnbP*) were selected from *B. breve* UCC2003, BLLJ_1505 (*lnbX*) was selected from *B. longum* subsp. *longum* JCM1217, and BBPR_1438 (*lnbB*) was selected from *B. bifidum* PRL2010.

Two signature genes, encoding glycoside hydrolases essential to the catabolic pathways of HMO central moieties LNT, LNnT and LNB, in *B. breve,* were identified based on the above results for *B. breve* UCC2003 (Supplemental Table S3). The *nahA* gene was identified as being crucial for the degradation of both LNT and LNnT (through the hydrolysis of lacto-N-triose), and *lnbP* was identified as essential for the utilisation of LNB. Additionally, one signature gene, *lnbB*, was identified in *B. bifidum* as encoding the key glycoside hydrolase required for the metabolism of LNT, based on previous literature^32^ (Supplemental Table S3). Another distinct glycoside hydrolase required for the metabolism of LNT in this same way, *lnbX*, was identified in a strain of *B. longum* subsp. *longum* by Sakurama *et al*.^37^, and thus was also selected (Supplemental Table S3). No genes were selected from *B. longum* subsp. *infantis*, as this species and *B. breve* appear to share the same functional homologs and thus appear to utilize the same pathways for the metabolism of LN(n)T and LNB. The deduced amino acid sequences of these four genes were employed as the reference sequences in a multiple alignment of all available *Bifidobacterium* genomes retrieved from the NCBI database, as described, and represented in a heatmap, based on a cut-off of 70% iterative similarity over 50% protein length, and an e-value of <0.0001 (Fig. 5). The representation obtained, ordered by origin of isolation, reveals the distribution of these key genes, and thus the metabolic pathways, required for LN(n)T/LNB utilisation across the *Bifidobacterium* genus. The *B. bifidum* gene *lnbB* and *B. longum* subsp. *longum* gene *lnbX* (whose products are responsible for the hydrolysis of LNT into LNB and lactose) appear to be individually unique to *B. bifidum* and (this species of) *B. longum* subsp. *longum*, respectively, with no clear homologs in any other species of *Bifidobacterium* including each other. The two *B. breve* signature genes used in the search yielded multiple significant hits for homologs, but to differing degrees. The analysis identified a relatively small number, i.e. four homologous *nahA* genes across the genus: one in the infant-associated species *B. longum* subsp. *infantis*, two in marmoset-associated and one tamarin-associated species- *B. callittrichos, B. ruteri*, and *B. saguini*, respectively. On the other hand, *lnbP* yielded 16 significant matches to homologous genes in other *Bifidobacterium* species, isolated from both human and non-human-related sources. The significant differences in the conservation of these genes across the genus are indicative of the importance of specific glycan moiety-utilising pathways in bifidobacteria.

## Discussion

**Figure 6:**
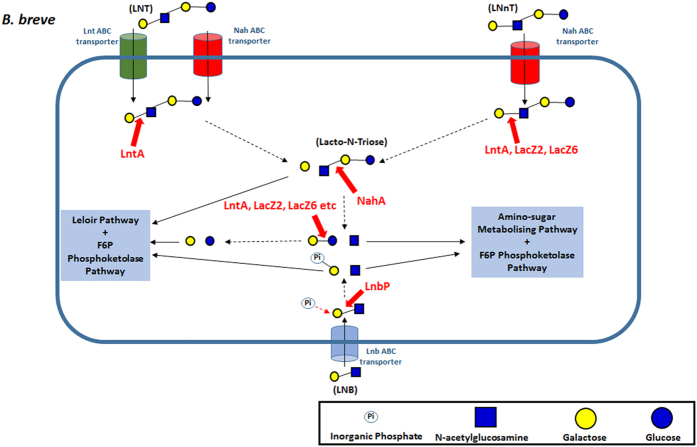
Schematic representation of the proposed model for the metabolism of free LNT, LNnT and LNB by *B. breve* UCC2003.

The role of HMOs as a selective substrate, for specific bacterial species in the neonatal gut, is now widely proposed as one of the key factors in the development of a healthy microbiota in early life. The high proportion of bifidobacteria, specifically the species *B. breve, B. longum* subsp. *infantis* and *B. bifidum*, in the microbiota of breastfed infants indicates their ability to utilise these carbohydrates as growth factors. Our findings allow us to propose a model for the utilisation of HMOs LN(n)T by *B. breve* (Fig. 6). In this model, LN(n)T is internalised by the cell and subsequently degraded by intracellular pathways into monosaccharides for energy production. While *B. breve* is able to metabolize smaller HMO components, such as fucose, fucosyllactose and sialic acid, which are released through extracellular hydrolysis of larger molecules^16^,^17^,^22^,^39^,^40^, our findings clearly show that *B. breve* can also utilise larger HMO structures. This expands our view of this gut commensal from being merely a scavenger, to an active and direct HMO utilizer.

Our multi-pronged approaches reveal the activities of individual components and thus the overall pathways that facilitate LNT and LNnT utilisation by *B. breve* UCC2003. LntA exhibits a triple specificity for the Galβ-1,3GlcNAc and Galβ-1,4GlcNAc linkages of LNT and LNnT, as well as the Galβ-1,4Glc moiety of lactose, as previously suggested^42^. However, the ability of UCC2003-lntA to grow on LNnT, but not on LNT, indicates that while LNT can only be intracellularly hydrolysed by LntA, the hydrolysis of LNnT is not exclusively attributable to this β-galactosidase, and can be degraded by other cellular glycoside hydrolases such as LacZ6 and LacZ2, releasing lacto-N-triose for hydrolysis by NahA, and galactose. It should be noted that both *lacZ2* and *lacZ6* have previously been shown to be involved in the metabolism of lactose and galacto-oligosaccharides^42^. Interestingly, these results mirror those previously shown by Yoshida *et al*.^29^, who, in *B. longum* subsp. *infantis* ATCC15697, demonstrated the preferential activities of one GH42 family glycoside hydrolase (Bga42A) in hydrolysing Type I HMOs and one GH2 family glycoside hydrolase (Bga2A) in hydrolysing Type II HMOs and lactose.

The transcriptomic results clearly implicate *nahS, lntS, lntP1* and *lntP2* in the utilisation on both LNT and LNnT, being involved in the internalisation of these sugars into the cell. The inability of the UCC2003-nahS mutant to grow on LNnT suggests the role of the *nah* locus-encoded transporter as the sole system responsible for LNnT internalisation by UCC2003. In contrast, since UCC2003-nahS displays growth on LNT comparable to that of the wild type strain, the *nah* transport system may not be involved in LNT transport, or may be, but with its function aided by one or more additional transport systems. The ability of the UCC2003-lntP1 and UCC2003-lntS to grow in mMRS supplemented with LNT demonstrates that the *lnt* transport system is also not exclusively, or potentially, at all, responsible for LNT internalisation. We therefore suggest that either these two transport systems may have at least partially overlapping substrate specificities, or that another yet undetermined transport system is partially, or wholly responsible for the internalisation of LNT.

While LNT and LNnT enter the *B. breve* cell as distinct isomers, their degradation products are identical and are shuttled through the same metabolic routes for energy production, i.e. two galactose molecules and one glucose to the Leloir and F6P phosphoketolase pathways and one GlcNAc to the amino-sugar metabolising pathway (thus both directly and indirectly feeding into the Bifid Shunt) (Fig. 6).

Although found within the molecular structure of LNT, free LNB is not released during the degradation of the Type I HMO central moiety by *B. breve* UCC2003. However, despite the relative low abundance of free LNB in human breast milk and thus the breastfed infant gut, *B. breve* UCC2003 possesses a distinct pathway for LNB utilisation. It has previously been suggested that LNB metabolism can be seen as a proxy for HMO utilisation by Bifidobacteria^17^,^28^, which explains the presence of this pathway in *B. breve*, which likely ‘sweeps up’ the free LNB released by the extracellular hydrolysis of larger HMO structures by other microbiota members, such as *B. bifidum*, and then utilise it via the GNB/LNB pathway.

The general model of LN(n)T and LNB utilisation in *B. breve* is mirrored by that found in *B. longum* subsp. *infantis*. The functional equivalent of *lacZ2* and *lacZ6* in *B. longum* subsp. *infantis* ATCC15697 is *bga2a*^29^. The ortholog of *lntA* is *bga42a*^29^, and the counterpart of *nahA* is *nagZ*^30^. A copy of *lnbP* is also found in *B. longum* subsp. *infantis* ATCC15697^31^. In contrast, HMO central moiety utilisation in *B. bifidum* diverges somewhat from the *B. breve*/*B. longum* subsp. *infantis* model. While *B. bifidum* possesses functionally equivalent orthologs of *lacZ2*/*lacZ6 (bbgIII*)^50^, and a *nahA (bbhI* and *bbhII*)^34^, all of these appear to be extracellular proteins, as opposed to the (predicted) intracellular localisation of their *B. breve* counterparts. However, the biggest defining factor separating the *B. breve*/*B. longum* subsp. *infantis* model and the *B. bifidum* model appears to be the hydrolysis pathway of Type I central moiety LNT. In *B. bifidum* PRL2010 LnbB hydrolyses LNT extracellularly at its GlcNAcβ1-3Gal linkage, releasing LNB, which is internalised, and phosphorolysed by *lnbP1* and *lnbP2*; and lactose, which is hydrolysed by β-galactosidase activities.

The absence of *B. bifidum lnbB* (and *B. longum* subsp. *longum* JCM1217 *lnbX*) homologs in other *Bifidobacterium* species highlights its unique function in HMO metabolism. This agrees with previous knowledge, as already mentioned, of *B. bifidum* (and one strain of *B. longum* subsp. *longum*) utilizing a significantly different pathway for LNT utilisation, and of HMO metabolism as compared to *B. breve* and *B. longum* subsp. *infantis*. A similarity search for the two *B. breve* LN(n)T/LNB signature genes, *nahA* and *lnbP*, among bifidobacteria shows the presence of homologs in various species of *Bifidobacterium*, but the extent of their conservation differs considerably. The sixteen species of *Bifidobacterium* that were shown to possess an *lnbP* homolog, have been isolated from a range of environments, including faecal samples of (both infant and adult) humans, primates and other mammals. The conservation of this gene across various bifidobacterial species points toward the importance of this gene in the GNB/LNB pathway, which is common to many *Bifidobacterium* species, and has previously been shown to function in roles such as mucin metabolism^28^,^46^,^51^,^52^. Interestingly, clear homologs of the *nahA* gene are only found in four other bifidobacterial species, the human isolate *B. longum* subsp. *infantis*, and three other primate-associated species *B. callitrichos* and *B. reuteri*, originally isolated from marmoset faeces, and *B. sanguini*, originally isolated from tamarin faeces. As LNT and similar oligosaccharide structures can be found in the glycome of primate milk^53^, the *nahA* homologs are expected to play a similar role in *Bifidobacterium* species associated with these hosts. This suggests a common adaptation of the LNT/LNnT-utilisation pathway among bifidobacteria associated with the primate gut, using their respective milk oligosaccharides as substrates, thereby explaining co-evolution with and colonisation of this host.

## Materials and Methods

### Bacterial strains, plasmids, and culture conditions

Bacterial strains and plasmids used in this study are listed in Supplemental Table S2. *B. breve* UCC2003 was routinely cultured in either de Man Rogosa and Sharpe medium (MRS medium; Difco, BD, Le Pont de Claix, France) supplemented with 0.05% cysteine-HCl or reinforced clostridial medium (RCM; Oxoid Ltd., Basingstoke, England). Carbohydrate utilization by bifidobacterial strains was examined in modified de Man Rogosa and Sharpe (mMRS) medium prepared from first principles^54^, and excluding a carbohydrate source. Prior to inoculation, the mMRS medium was supplemented with cysteine-HCl (0.05%, wt/vol) and a particular carbohydrate source (1%, wt/vol). It has previously been shown that mMRS does not support growth of *B. breve* UCC2003 in the absence of an added carbohydrate^55^. Carbohydrates used were lactose (Sigma Aldrich, Steinheim, Germany), LNB (Elicityl Oligotech, Crolles, France), lactosamine-hydrochloride (lactosamine-HCl) (Glycom, Lyngby, Denmark), LNT (Glycom, Lyngby, Denmark; Elicityl Oligotech, Crolles, France) and LNnT (Glycom, Lyngby, Denmark). A 1% wt/vol concentration of carbohydrate was considered sufficient to analyse the growth capabilities of a strain on a particular carbon source. The addition of these carbohydrates did not significantly alter the pH of the medium, and therefore subsequent pH adjustment was not required.

*B. breve* cultures were incubated under anaerobic conditions in a modular atmosphere-controlled system (Davidson and Hardy, Belfast, Ireland) at 37 °C. *Lactococcus lactis* strains were cultivated in M17 broth (Oxoid Ltd., Basingstoke, England) containing 0.5% glucose^56^ at 30 °C. *Escherichia coli* strains were cultured in Luria-Bertani (LB) broth^57^ at 37 °C with agitation. Where appropriate, growth media contained tetracycline (Tet; 10 μg ml^−1^), chloramphenicol (Cm; 5 μg ml^−1^ for *L. lactis* and *E. coli*, 2.5 μg ml^−1^ for *B. breve*), erythromycin (Em; 100 μg ml^−1^) or kanamycin (Kan; 50 μg ml^−1^). Recombinant *E. coli* cells containing (derivatives of) pORI19 were selected on LB agar containing Em and Kan, and supplemented with X-gal (5-bromo-4-chloro-3-indolyl-β-D-galactopyranoside) (40 μg ml^−1^) and 1 mM IPTG (isopropyl-β-D-galactopyranoside). In order to determine bacterial growth profiles and final optical densities, 5 ml of freshly prepared mMRS medium, including a particular carbohydrate (see above), was inoculated with 50 μl (1%) of a stationary phase culture of *B. breve* UCC2003. Uninoculated mMRS medium was used as a negative control. Cultures were incubated anaerobically at 37 °C for 24 h, and the optical density at 600 nm (OD600) was determined manually, or using a PowerWave microplate spectrophotometer (BioTek Instruments, Inc., USA) in conjunction with Gen5 microplate software for Windows, at the end of this period, as described previously^55^,^58^.

### Bifidobacterium breve Growth Assays

Growth profiles of sixteen distinct *Bifidobacterium breve* strains from the UCC collection (listed in Supplemental Table S2) on LNT or LNnT, as the sole carbohydrate source, using lactose as a positive control, were determined in mMRS, using the microplate spectrophotometer, as described above. LNB and lactosamine-HCl were not included in these assays, as sufficient (and affordable) quantities of these carbohydrate substrates could not be obtained.

Growth profiles of insertion mutant, Tn5 transposon mutant, and complementation strains of *B. breve* UCC2003 generated in this and other studies, were determined, manually, as described above, adopting LNT, LNnT or LNB as carbohydrate source and in each case using lactose as a positive control.

### Nucleotide sequence analysis

Sequence data were obtained from the Artemis-mediated^59^ genome annotations of *B. breve* UCC2003^60^. Database searches were performed using non-redundant sequences accessible at the National Center for Biotechnology Information (NCBI) (http://www.ncbi.nlm.nih.gov) using the basic local alignment search tool (BLAST)^61^,^62^. Sequences were verified and analysed using the SeqMan and SeqBuilder programs of the DNAStar software package (version 10.1.2; DNAStar, Madison, WI, USA). Gene product (protein) localisation and signal peptide predictions were made using the TMHMM, v. 2.0 and SignalP, v. 4.1^63^ servers, respectively, available at http://www.cbs.dtu.dk/.

### DNA Manipulations

Chromosomal DNA was isolated from *B. breve* UCC2003 as previously described^64^. Plasmid DNA was isolated from *E. coli, L. lactis* and *B. breve* using the Roche High Pure Plasmid Isolation kit (Roche Diagnostics, Basel, Switzerland). An initial lysis step was performed using 30 mg ml^−1^ of lysozyme for 30 minutes at 37 °C prior to plasmid isolation from *L. lactis* or *B. breve*. DNA manipulations were essentially performed as described previously^57^. All restriction enzymes and T4 DNA ligase were used according to the supplier’s instructions (Roche Diagnostics, Basel, Switzerland). Synthetic single stranded oligonucleotide primers used in this study (Supplemental Table S1) were synthesized by Eurofins (Ebersberg, Germany). Standard PCRs were performed using Taq PCRmaster mix (Qiagen) or Extensor Hi-Fidelity PCR Master Mix (Thermo Scientific, Waltham, United States) in a Life Technologies Proflex PCR System (Thermo Scientific, Waltham, United States). PCR products were visualized by ethidium bromide (EtBr) staining following agarose gel electrophoresis (1% agarose). *B. breve* colony PCR reactions were performed as described previously^65^. PCR fragments were purified using the Roche high Pure PCR purification kit (Roche Diagnostics, Basel, Switzerland). Plasmid DNA was isolated using the Roche High Pure Plasmid Isolation kit (Roche Diagnostics, Basel, Switzerland). Plasmid DNA was introduced into *E. coli* by electroporation as described previously^57^. *B. breve* UCC2003^66^ and *L. lactis*^67^ were transformed by electroporation according to published protocols. The correct orientation and integrity of all plasmid constructs (see also below) were verified by DNA sequencing, performed at Eurofins (Ebersberg, Germany).

### Analysis of global gene expression using *B. breve* DNA microarrays

Global gene expression was determined during log-phase growth of *B. breve* UCC2003 in mMRS supplemented with either LNT, LNnT, LNB, lactosamine-HCl or lactose. The obtained transcriptome was compared to that determined for log-phase *B. breve* UCC2003 cells when grown in mMRS supplemented with ribose. DNA microarrays containing oligonucleotide primers representing each of the 1864 identified open reading frames on the genome of *B. breve* UCC2003 were designed and obtained from Agilent Technologies (Palo Alto, Ca., USA). Methods for cell disruption, RNA isolation, RNA quality control, complementary DNA synthesis and labelling were performed as described previously^68^. Labelled cDNA was hybridized using the Agilent Gene Expression hybridization kit (part number 5188-5242) as described in the Agilent Two-Colour Microarray-Based Gene Expression Analysis v4.0 manual (publication number G4140-90050). Following hybridization, microarrays were washed in accordance with Agilent’s standard procedures and scanned using an Agilent DNA microarray scanner (model G2565A). Generated scans were converted to data files with Agilent’s Feature Extraction software (Version 9.5). DNA-microarray data were processed as previously described^69^,^70^,^71^. Differential expression tests were performed with the Cyber-T implementation of a variant of the t-test^72^.

### Construction of *B. breve* UCC2003 insertion mutants

An internal fragment of Bbr_0530 (designated here as *lntS*) (465 bp, representing codon numbers 61 through to 216 of the 420 codons of this gene), Bbr_1554 (designated here as *nahS*) (488 bp, representing codon numbers 92 through to 255 of the 442 codons of this gene), and Bbr_1556 (designated here as *nahA*) (443 bp, representing codon numbers 70 through to 218 of the 660 codons of this gene) were amplified by PCR using *B. breve* UCC2003 chromosomal DNA as a template and primer pairs IM530F and IM530R, IM1554F and IM1554R, or IM1556F and IM1556R, respectively (Supplemental Table S1). The insertion mutants were constructed using a previously described approach^65^. Site-specific recombination of potential tet-resistant mutant isolates was confirmed by colony PCR using primer combinations tetWFw and tetWRv to verify *tetW* gene integration, and primers 530confirm1 or 530confirm2, 1554Confirm1 or 1554Confirm2, and 1556confirm1 or 1556confirm2 (positioned upstream of the selected internal fragments of Bbr_0530, Bbr_1554 and Bbr_1556, respectively) in combination with primer tetWFw to confirm integration at the correct chromosomal location (Supplemental Table S1).

### Complementation of *B. breve* insertion mutants

DNA fragments encompassing Bbr_0529 (designated here as *lntA*), Bbr_1554 (*nahS*) and Bbr_1556 (*nahA*) were generated by PCR amplification from *B. breve* UCC2003 chromosomal DNA using Q5 High-Fidelity Polymerase (New England BioLabs, Herefordshire, United Kingdom) and primer pairs: 529pNZ44F and 529pNZ44R, 1554PCB1.2F and 1554PBC1.2R, and 1556PBC1.2F and 1556PBC1.2R, respectively (Supplemental Table S1).

The resulting *lntA*-encompassing fragment was digested with PstI and XbaI, and ligated to the similarly digested pNZ44^73^. The ligation mixture was introduced into *L. lactis* NZ9000 by electrotransformation and transformants were then selected based on chloramphenicol resistance. The plasmid content of a number of Cm-resistant transformants was screened by restriction analysis. The integrity of the cloned insert of one of the recombinant plasmids, designated pNZ44-lntA, was confirmed by sequencing. The *lntA*-coding sequence, together with the constitutive p44 lactococcal promoter, specified by pNZ44, was amplified by PCR from pNZ44-lntA using Q5 High-Fidelity DNA polymerase and primer combination P44 Forward and 529pNZ44R (Supplemental Table S1). The resulting DNA fragment was digested with EcoRV and XbaI, and ligated to the similarly digested pBC1.2^74^, generating pBC1.2-lntA.

PCR-generated DNA fragments encompassing *nahS* and *nahA*, including their presumed promoter regions, were digested with BamHI and XbaI, and ligated to the similarly digested pBC1.2 to generate pBC1.2-nahS or pBC1.2-nahA, respectively. The ligation mixtures were introduced into *E. coli* XL1-blue by electrotransformation and transformants selected based on tetracycline and chloramphenicol resistance. Transformants were checked for plasmid content using colony PCR, restriction analysis of plasmid DNA, and verified by sequencing. Plasmids pBC1.2-lntA, pBC1.2-nahS or pBC1.2-nahA were introduced into the insertion mutant *B. breve* UCC2003-lntA, *B. breve* UCC2003-nahS and *B. breve* UCC2003-nahA^42^, respectively, by electrotransformation and transformants were selected based on tetracycline and chloramphenicol resistance.

### Construction of overexpression vectors, protein overproduction and purification

For the construction of the plasmid pNZ-nahA, a DNA fragment encompassing the predicted N-acetylhexosaminidase-encoding gene *nahA* was generated by PCR amplification from chromosomal DNA of *B. breve* UCC2003 using Q5 High-Fidelity DNA polymerase and the primer combination 1556F and 1556R (Supplemental Table S1). An in-frame N-terminal His10-encoding sequence was incorporated into the forward primer 1556F to facilitate downstream protein purification. The generated amplicons were digested with PvuII and XbaI, and ligated into the ScaI and XbaI-digested, nisin-inducible translational fusion plasmid pNZ8150^75^. The ligation mixtures were introduced into *L. lactis* NZ9000 by electrotransformation and transformants were then selected based on chloramphenicol resistance. The plasmid content of a number of Cm-resistant transformants was screened by restriction analysis and the integrity of positively identified clones was verified by sequencing.

Nisin-inducible gene expression and protein overproduction was performed as described previously^76^,^77^,^78^. In brief, 400 ml of M17 broth supplemented with 0.5% (wt/vol) glucose was inoculated with a 2% inoculum of a particular *L. lactis* strain, followed by incubation at 30 °C until an OD600 of 0.5 was reached, at which point protein expression was induced by addition of cell-free supernatant of a nisin-producing strain^79^, followed by continued incubation for a further 2 hours. Cells were harvested by centrifugation and protein purification achieved as described previously^76^. Protein concentrations were determined using the Bradford method^80^.

### Assay of individual and combined β-Galactosidase activities

The individual or sequential hydrolytic activities specified by *lntA* (corresponding to Bbr_0529), *nahA* (corresponding to Bbr_1556), *lacZ2* (corresponding to Bbr_0010) and *lacZ6* (corrsponding to Bbr_1552) were determined essentially as described previously^78^, using LNT, LNnT or lactose as a substrate. Briefly, a 50-μl volume of each purified protein (protein concentration of 0.5 mg/ml) was added to 20 mM morpholinepropanesulfonic acid (MOPS) (pH 7.0) buffer and 1 mg ml^−1^ (wt/vol) of one of the above-mentioned sugars in a final volume of 1 ml, followed by incubation for 24 hours at 37 °C. When sequential activities were assessed, a sample was heated to 85 °C for 15 minutes following 12 hour incubation with the first enzyme and a given substrate, before the addition of the addition of a second enzyme, which was then followed by a further 12-hour incubation at 37 °C. All samples were subject to a final enzyme denaturation step at 85 °C for 15 minutes, before storage at −20 °C.

### HPAEC-PAD analysis

For HPAEC-PAD analysis, a Dionex (Sunnyvale, CA) ICS-3000 system was used. Carbohydrate fractions from the above-mentioned hydrolysis assays (25 μl aliquots) were separated on a CarboPac PA1 analytical-exchange column (dimensions, 250 mm by 4 mm) with a CarboPac PA1 guard column (dimensions, 50 mm by 4 mm) and a pulsed electrochemical detector (ED40) in PAD mode (Dionex). Elution was performed at a constant flow-rate of 1.0 ml/min at 30 °C using the following eluents for the analysis: eluent A, 200 mM NaOH; eluent B, 100 mM NaOH plus 550 mM Na acetate; eluent C, Milli-Q water. The following linear gradient of sodium acetate was used with 100 mM NaOH: from 0 to 50 min, 0 mM; from 50 to 51 min, 16 mM; from 51 to 56 min, 100 mM; from 56 to 61 min, 0 mM. Chromatographic profiles of standard carbohydrates were used for comparison of the results of their breakdown by LntA, LacZ2, LacZ6 and NahA proteins. Chromeleon software (version 6.70; Dionex Corporation) was used for the integration and evaluation of the chromatograms obtained. A 1 mg/ml stock solution of each of the carbohydrates, as well as their putative breakdown products (where available) used as reference standards was prepared by dissolving the particular sugar in Milli-Q water.

### Bioinformatic Analysis

Based on the analysis of the microarray results and functional characterisation of gene loci from *B. breve* UCC2003, as well as previously published data on HMO utilisation by *B. longum* subsp. *infantis* and *B. bifidum* and *B. longum* subsp. *longum*^18^,^28^,^29^,^30^,^31^,^32^,^33^,^34^,^35^,^36^,^37^, four genes were identified as crucial for the utilisation of Type I central moieties LNT and LNB, and Type II HMO moiety LNnT. On-line available genomic data sets of bifidobacteria were first retrieved from the NCBI website (http://www.ncbi.nlm.nih.gov) and aligned using an all-vs-all BLASTP approach^61^, using 70% of iterative similarity across all available *Bifidobacterium* species over 50% of protein length and a 0.0001 e-value as a significance cut-off. The resulting alignment was subsequently clustered in MCL families of orthologous genes using the mclblastline algorithm^81^. The resulting output was used to first build a presence/absence binary matrix, and then the genes of interest were selected and represented in a heatmap employing a code colour grading that represents the degree of sequence similarity, with species ordered by origin of isolation. Bbr_1556 (*nahA*) and Bbr_1587 (*lnbP*) were selected from *B. breve* UCC2003, BLLJ_1505 (*lnbX*) from *B. longum* subsp. *longum* JCM1217 and BBPR_1438 (*lnbB*) was selected from *B. bifidum* PRL2010.

### Microarray data accession number

The microarray data obtained in this study have been deposited in NCBI’s Gene Expression Omnibus database and are accessible through GEO Series accession number GSE84710.

## Additional Information

**How to cite this article**: James, K. *et al*. *Bifidobacterium breve* UCC2003 metabolises the human milk oligosaccharides lacto-N-tetraose and lacto-N-neo-tetraose through overlapping, yet distinct pathways. *Sci. Rep.* **6**, 38560; doi: 10.1038/srep38560 (2016).

**Publisher’s note:** Springer Nature remains neutral with regard to jurisdictional claims in published maps and institutional affiliations.

## Supplementary Material

Supplementary Information

## Figures and Tables

**Figure 1:**
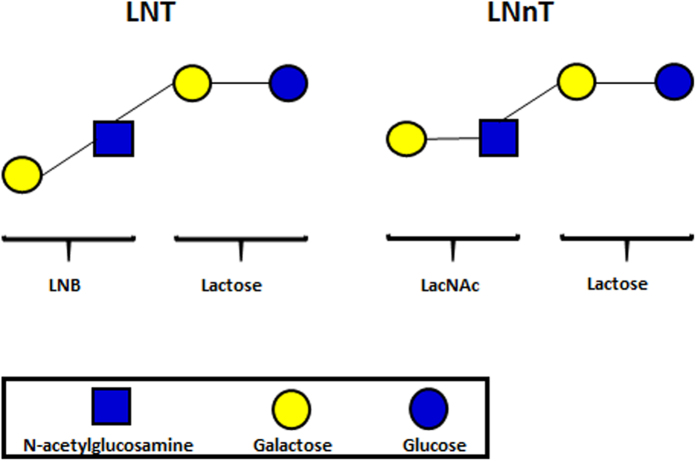
Schematic structures of Type I HMO moiety LNT, and Type II HMO moiety LNnT.

**Table 1:** *B. breve* UCC2003 genes upregulated in expression during growth in mMRS medium supplemented with 1% LNT, LNnT, LNB, lactosamine-HCl, or lactose as the sole carbohydrate.

Gene ID	Gene name	Function	Fold upregulation^a,b^ during growth on:				
			LNT	LNnT	LNB	Lactosamine-HCl	Lactose
Bbr_0417	galC	Solute-binding protein of ABC transporter system for sugars	—	—	—	10.93	—
Bbr_0418	galD	Permease protein of ABC transporter system for sugars	3.23	5.18	—	6.98	—
Bbr_0419	galE	Permease protein of ABC transporter system for sugars	3.10	7.88	3.18	9.80	3.07
Bbr_0420	galG	GH42 lacZ4 Beta-galactosidase	—	—	—	3.85	—
Bbr_0421	galR	Transcriptional regulator, LacI family	—	—	—	—	—
Bbr_0422	galA	GH53 galA Endogalactanase	—	—	—	4.07	—
Bbr_0490	Bbr_0490	Transcriptional regulator, DeoR family	—	3.27	—	2.60	—
Bbr_0491	galT	Galactose-1-phosphate uridylyltransferase	—	—	5.08	—	—
Bbr_0492	galK	Galactokinase	—	—	3.57	—	—
**Bbr_0526**	**lntR**	**Transcriptional regulator, LacI family**	—	**3.95**	—	**10.16**	—
**Bbr_0527**	**lntP1**	**Permease protein of ABC transporter system for sugars**	**6.81**	**10.05**	—	**8.57**	**3.48**
**Bbr_0528**	**lntP2**	**Permease protein of ABC transporter system for sugars**	**3.23**	**10.64**	**3.03**	**11.15**	**3.35**
**Bbr_0529**	**lntA**	**GH42 Beta-galactosidase**	**6.61**	**12.58**	—	**9.53**	**3.64**
**Bbr_0530**	**lntS**	**Solute-binding protein of ABC transporter system for sugars**	**4.15**	**5.20**	**2.97**	**17.17**	**3.31**
Bbr_0845	glgP2	glgP2 Glycogen phosphorylase	—	—	—	2.73	—
Bbr_0846	nagA1	nagA1 N-acetylglucosamine-6-phosphate deacetylase	—	—	—	4.76	—
Bbr_0847	nagB2	nagB2 Glucosamine-6-phosphate isomerase	—	—	—	5.82	—
Bbr_0848	Bbr_0848	Sugar kinase, ROK family	3.30	5.12	—	10.69	—
Bbr_0849	Bbr_0849	NagC/XylR-type transciptional regulator	—	2.90	—	14.40	—
Bbr_0850	Bbr_0850	Aldose 1-epimerase family protein	—	—	—	7.39	—
Bbr_0851	Bbr_0851	Glucose/fructose transport protein	2.91	3.75	—	16.43	—
Bbr_0852	atsA2	Sulfatase family protein	—	—	—	4.18	—
Bbr_0853	atsB2	atsB Arylsulfatase regulator (Fe-S oxidoreductase)	—	—	—	2.70	—
Bbr_0854	Bbr_0854	Conserved hypothetical membrane spanning protein with DUF81 domain	—	—	—	4.03	—
Bbr_0855	Bbr_0855	Hypothetical protein	—	—	—	7.31	—
Bbr_0856	Bbr_0856	Conserved hypothetical membrane spanning protein	—	—	—	3.82	—
Bbr_1247	nagA2	CE9 nagA2 N-acetylglucosamine-6-phosphate deacetylase	2.67	5.06	3.92	—	—
Bbr_1248	nagB3	nagB3 Glucosamine-6-phosphate isomerase	4.11	5.95	8.51	—	—
Bbr_1249	Bbr_1249	Transcriptional regulator, ROK family	—	—	—	3.84	—
Bbr_1250	Bbr_1250	Sugar kinase, ROK family	2.44	7.70	—	8.75	—
Bbr_1251	Bbr_1251	N-acetylglucosamine repressor	—	—	—	7.71	—
Bbr_1252	pfkB	Fructokinase	—	—	—	—	—
Bbr_1550	Bbr_1550	Hypothetical protein	—	2.90	—	28.72	2.81
**Bbr_1551**	**lacS**	**Galactoside symporter**	**13.10**	**73.82**	—	**43.73**	**31.62**
**Bbr_1552**	**LacZ6**	**GH2 Beta-galactosidase**	**44.01**	**105.71**	—	**39.65**	**11.01**
**Bbr_1553**	**lacI**	**Transcriptional regulator, LacI family**	—	**4.56**	—	**15.60**	—
**Bbr_1554**	**nahS**	**Solute-binding protein of ABC transporter system (lactose)**	**5.71**	**15.05**	**9.91**	**13.10**	—
**Bbr_1555**	**nahR**	**NagC/XylR-type transciptional regulator**	**4.86**	**12.53**	—	**21.74**	—
**Bbr_1556**	**nahA**	**GH20 nagZ Beta-N-acetylhexosaminidase**	**2.65**	**3.74**	—	**2.90**	—
**Bbr_1558**	**nahP**	**Permease protein of ABC transporter system**	—	—	**4.97**	—	—
**Bbr_1559**	**nahT1**	**ATP-binding protein of ABC transporter system**	—	—	—	**2.66**	—
**Bbr_1560**	**nahT2**	**ATP-binding protein of ABC transporter system**	—	—	**3.42**	**2.51**	—
**Bbr_1585**	**galE**	**UDP-glucose 4-epimerase**	—	**3.11**	**3.33**	**4.12**	—
**Bbr_1586**	**nahK**	**Phosphotransferase family protein**	**4.55**	**10.69**	**3.19**	—	—
**Bbr_1587**	**lnbP**	**GH112 lacto-N-biose phorylase**	—	**3.86**	**6.52**	—	—
**Bbr_1588**	**galP1**	**Permease protein of ABC transporter system for sugars**	**3.38**	**4.20**	**6.38**	—	—
**Bbr_1589**	**galP2**	**Permease protein of ABC transporter system for sugars**	**2.78**	**5.47**	**4.05**	—	—
**Bbr_1590**	**galS**	**Solute-binding protein of ABC transporter system for sugars**	**4.41**	**4.45**	**16.49**	—	—

The level of expression is shown as a fold-value of increase in expression on each carbohydrate, as compared to a ribose control, with a cut-off of a minimum 2.5-fold increase in expression. Genes within the 4 loci focused on in this study are shown in bold script.

^a^Based on comparative transcriptome analysis using B. breve UCC2003 grown on 1% LNT, LNnT, LNB, lactosamine-HCl or lactose compared to growth on ribose. Microarray data were obtained using B. breve UCC2003 grown on 1% LNT, LNnT, LNB, lactosamine-HCl or lactose and were compared with array data obtained when B. breve UCC2003 was grown on ribose as a control.

^b^The cutoff point is 2.5-fold, with a P value of _0.001. —, value below the cutoff.
