# Supplementary material for: Bifidobacterium breve UCC2003 metabolises the human milk oligosaccharides lacto-N-tetraose and lacto-N-neo-tetraose through overlapping, yet distinct pathways
Source: Sci Rep. 2016 Dec 8;6:38560. doi: 10.1038/srep38560 (PMC5144078; doi:10.1038/srep38560)
Supplement: Supplementary Information [file srep38560-s1.pdf]

Title: *Bifidobacterium breve* UCC2003 metabolises the human milk oligosaccharides lacto-N-tetraose and lacto-N-neo-tetraose through overlapping, yet distinct pathways.

Authors: Kieran James[1,2], Mary O'Connell Motherway[2], Francesca Bottacini[2], Douwe van Sinderen[1,2*]

Affiliation(s): [1] School of Microbiology & [2]APC Microbiome Institute,

University College Cork, Cork, Ireland

*** Correspondence:** d.vansinderen@ucc.ie

Purpose	Primer	Sequence (5'-3')
Cloning of Bbr_1556 in pNZ8150	1556F	tgcatc**cagctg**atg*catcaccatcaccatcaccatcaccatcac*aacggtcaaacatctctgcaa
	1556R	tgcgca**tctaga**ggattggaagcgcagatggtc
Cloning of internal 465bp fragment of Bbr_0530 in pORI19	IM530F	ctggtc**aagctt**gaaggagaccggcatcaagg
	IM530R	ctggtc**tctaga**gtctggaacggcttggcgc
Cloning of internal 488bp fragment of Bbr_1554 in pORI19	IM1554F	ctggtc**aagctt**gaaggagaccggcatcaagg
	IM1554R	ctggtc**tctaga**gtctggaacggcttggcgc
Cloning of internal 443bp fragment of Bbr_1556 in pORI19	IM1556F	ctggtc**aagctt**cgaatacctcgccatccc
	IM1556R	ctggtc**tctaga**ccagtgacttcatccagtcgtc
Amplification of *tet*W	tetWFw	tcagctgtcgacatgctcatgtacggtaaggaagca
	tetWRv	gcgacggtcgaccataacttctgattgttgccg
Confirmation of site specific homologous recombination	526confirm1	gcgctagctgttacaatggtc
	526confirm2	gccatttccaacccctctc
	530confirm1	gaggaaggagaacagaacgatg
	530confirm2	gcgaaaggaatggcttcatag
	1554confrim1	ctgcgacgccaatgccgacaag
	1554confrim2	ccgctactgcaggtcgccatcatcaagcac
	1556confrim1	gcagctcatcgacgacatccgc
	1556confrim2	gcgcgcgcagtcggtgac
Cloning of 2086bp fragment containing Bbr_0529 into pNZ44	529pNZ44F	ctggtc**ctgcag**gactcccatttgcccacacgtag
	529pNZ44R	cgagc**tctaga**gatttctcctcgacactg
Cloning of p44-529 in pBC1.2	P44 Forward	ctggtcc**ggatcc**gagataatgccgactgtac
Cloning of 1637bp fragment containing Bbr_1554 into PBC1.2	1554PBC1.2F	ctggtccggatccgccggaacaacagtctgc
	1554PBC1.2R	cgagctctagaattcaccgtttggattattgg
Cloning of 2360bp fragment containing Bbr_1556 into PBC1.2	1556PBC1.2F	ctggtccggatccccacgcaggtggacaagt
	1556PBC1.2R	cgagctctagagaaatctcacaatcgcagcc

Restriction sites incorporated into oligonucleotide primer sequences are indicated in bold, and His-tag sequences incorporated into nucleotide primer sequences are indicated in italics.

Supplementary Table S1: Oligonucleotide primers used in this work.

Strain or plasmid	Relevant Features	Reference or Source
**Strains**		
Escherichia coli strains		
E. coli EC101	Cloning host, repA$^+$ km$^+$	[1]
E. coli EC101-pNZ-M.BbrII + M.BbrIII	EC101 harbouring pNZ8048 derivative containing *bbrIIM* and *bbrIIIM*	[2]
E. coli XL1-Blue	*supE44 hsdR17 recA1 gyrA96 thi relA1 lac* F' [*proAB+ lacI*q *lacZΔM15* Tn*10*(Tet^r)]	Stratagene
E.coli XL1-blue-pBC1.2-529	XL1-blue containing pBC1.2-529	This study
Lactococcus lactis strains		
L. lactis NZ9000	MG1363, pepN::nisRK, nisin-inducible overexpression host	[3]
L. lactis NZ9700	Nisin-producing strain	[3]
L. lactis NZ9000-pNZ-lacZ2	NZ9000 conatining pNZ-010	[4]
L. lactis NZ9000-pNZ-lntA	NZ9000 conatining pNZ-529	[4]
L. lactis NZ9000-pNZ-lacZ6	NZ9000 conatining pNZ-1552	[4]
L. lactis NZ9000-pNZ-nahA	NZ9000 conatining pNZ-1556	This study
L. lactis NZ9000-pNZ44-lntA	NZ9000 containing pNZ44-529	This study
Bifidobacterium sp. Strains		
B. breve UCC2003	Isolate from nursling stool	[5]
B. breve UCC2003-lacZ2	Tn5-transposon mutant in Bbr_0010 of UCC2003	[6]
B. breve UCC2003-lntP1	pORI19-tet-bbr_0527 insertion mutant of UCC2003	[4]
B. breve UCC2003-lntA	pORI19-tet-bbr_0529 insertion mutant of UCC2003	[4]
B. breve UCC2003-lacS	pORI19-tet-bbr_1551 insertion mutant of UCC2003	[4]
B. breve UCC2003-lacZ6	pORI19-tet-bbr_1552 insertion mutant of UCC2003	[4]
B. breve UCC2003-lnbP	pORI19-tet-bbr_1587 insertion mutant of UCC2003	[7]
B. breve UCC2003-lntS	pORI19-tet-bbr_0530 insertion mutant of UCC2003	This study
B. breve UCC2003-nahS	pORI19-tet-bbr_1554 insertion mutant of UCC2003	This study
B. breve UCC2003-nahA	pORI19-tet-bbr_1556 insertion mutant of UCC2003	This study
B. breve UCC2003-lntA-PBC1.2-lntA	pORI19-tet-529 insertion mutant of UCC2003 harbouring complementation construct pBC1.2-529	This study
B. breve UCC2003-nahS-PBC1.2-nahS	pORI19-tet-1554 insertion mutant of UCC2003 harbouring complementation construct pBC1.2-1554	This study
B. breve UCC2003-nahA-PBC1.2-nahA	pORI19-tet-1556 insertion mutant of UCC2003 harbouring complementation construct pBC1.2-1556	This study
B. breve UCC2004	Isolate from infant faeces	UCC
B. breve JCM7017	Isolate from human faeces	JCM
B. breve JCM7019	Isolate from infant faeces	JCM
B. breve NCFB2258	Isolate from infant intestine	NCFB
B. breve UCC2005	Isolate from nursling stool	UCC
B. breve 8815	Isolate from human faeces	NCIMB
B. breve 11815	Isolate from infant intestine	NCIMB
B. breve NIZO658	Isolate from human faeces	NIZO
B. breve UCC2006	Mother's milk	UCC
B. breve UCC2007	Mother's milk	UCC
B. breve UCC2008	Isolate from infant/adult faeces	UCC
B. breve LMG13208	Isolate from infant faeces	LMG
B. breve UCC2009	Isolate from infant/adult faeces	UCC
B. breve UCC2010	Mother's milk	UCC
B. breve UCC2011	Mother's milk	UCC
**Plasmids**		
pAM5	pBC1-puC19-Tet^r	[8]
pORI19	Em^r, repA$^-$, ori$^+$, cloning vector	[1]
pORI19-tet-lntS	Internal 465 bp fragment of *bbr_0530* and tetW cloned in pORI19	This study
pORI19-tet-nahS	Internal 443 bp fragment of *bbr_1554* and tetW cloned in pORI19	This study
pORI19-tet-nahA	Internal 443 bp fragment of *bbr_1556* and tetW cloned in pORI19	This study
pNZ8150	Cm^r, nisin inducible translational fusion vector	[9]
pNZ-nahA	Cm^r, pNZ8150 derivative containing translational fusion of Bbr_1556 encoding DNA fragment to nisin inducible promoter	This study
pNZ44	pNZ8048 containing constitutive p44 promoter from Lactococcal chromosome	[10]
pNZ44-lntA	pNZ44 harbouring Bbr_0529 downstream of p44 promoter	This study
pBC1.2	pBC1-pSC101-Cm^r	[8]
pBC1.2-lntA	pBC1-pSC101-Cmr harbouring Bbr_0529 downstream of p44 promoter	This study
pBC1.2-nahS	pBC1-pSC101-Cmr harbouring Bbr_1554	This study
pBC1.2-nahA	pBC1-pSC101-Cmr harbouring Bbr_1556	This study

Cm^r, Em^r,Km^r and Tet^r, resistance to chloramphenicol, erythromycin, kanamycin and tetracycline, respectively.

Collection of the University of Goteborg**;** CIP**,** Collection de l'Institut Pasteur; DSM**,** German Collection of Microorganisms and Cell Cultures; JCM, Japan Collection of Microorganisms; LMG, Belgian Co-ordinated Collection of Microorganisms; NCDO, National Collection of Dairy Organisms; NCFB, National Collection of Food Bacteria; NCIMB, National Collection of Industrial and Marine Bacteria; NCTC, National Collection of Type Cultures; UCC, University College Cork Culture Collection.

Supplementary Table S2: Bacterial plasmids and strains used in this work.

Gene ID	Gene Name	Location of Activity	Function in HMO Metabolism
***Bifidobacterium breve* UCC2003**			
Bbr_1556	nahA	Intracellular	nagZ Beta-N-acetylhexosaminidase; hydrolyses Lacto-N-Triose at GlcNAcβ1-3Gal residue, releasing GlcNAc and galactose.
Bbr_1587	lnbP	Intracellular	Lacto-N-biose phosphorylase; hydrolyses LNB, releasing GlcNAc and phosphorylated galactose.
***Bifidobacterium bifidum* PRL2010**			
BBPR_1438	lnbB	Extracellular	Lacto-N-biosidase; hydrolyses LNT at its GlcNAcβ1-3Gal residue, releasing LNB and lactose.
***Bifidobacterium longum* subsp. *longum* JCM1217**			
BLLJ_1505	lnbX	Extracellular	Lacto-N-biosidase; hydrolyses LNT at its GlcNAcβ1-3Gal residue, releasing LNB and lactose.

Supplementary Table S3: Locus tags, names and product functions of *B. breve* UCC2003 and *B. bifidum* PRL2010 genes selected for bioinformatic analysis of LN(n)T/LNB utilisation-associated homologs across the *Bifidobacterium* genus.

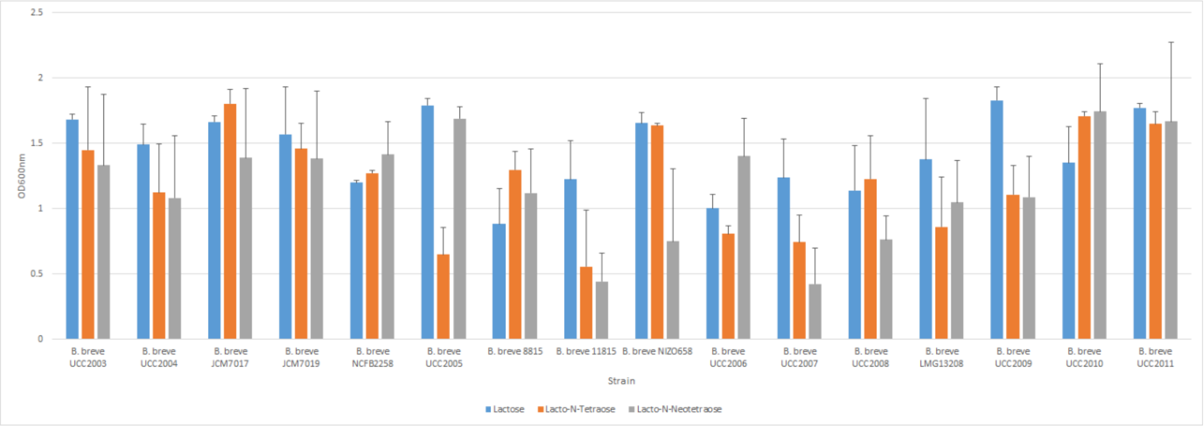

Supplementary Figure S1: Final OD_{600nm} values (measured using PowerWave microplate spectrophotometer) after 24 hours of growth of 16 *B. breve* strains in modified MRS containing 1 % (wt/vol) LNT, 1 % (wt/vol) LNnT or 1 % (wt/vol) lactose as the sole carbon source.

The results are the mean values obtained manually from two separate experiments (due to the limited availability of certain carbohydrates). Error bars represent the standard deviation.

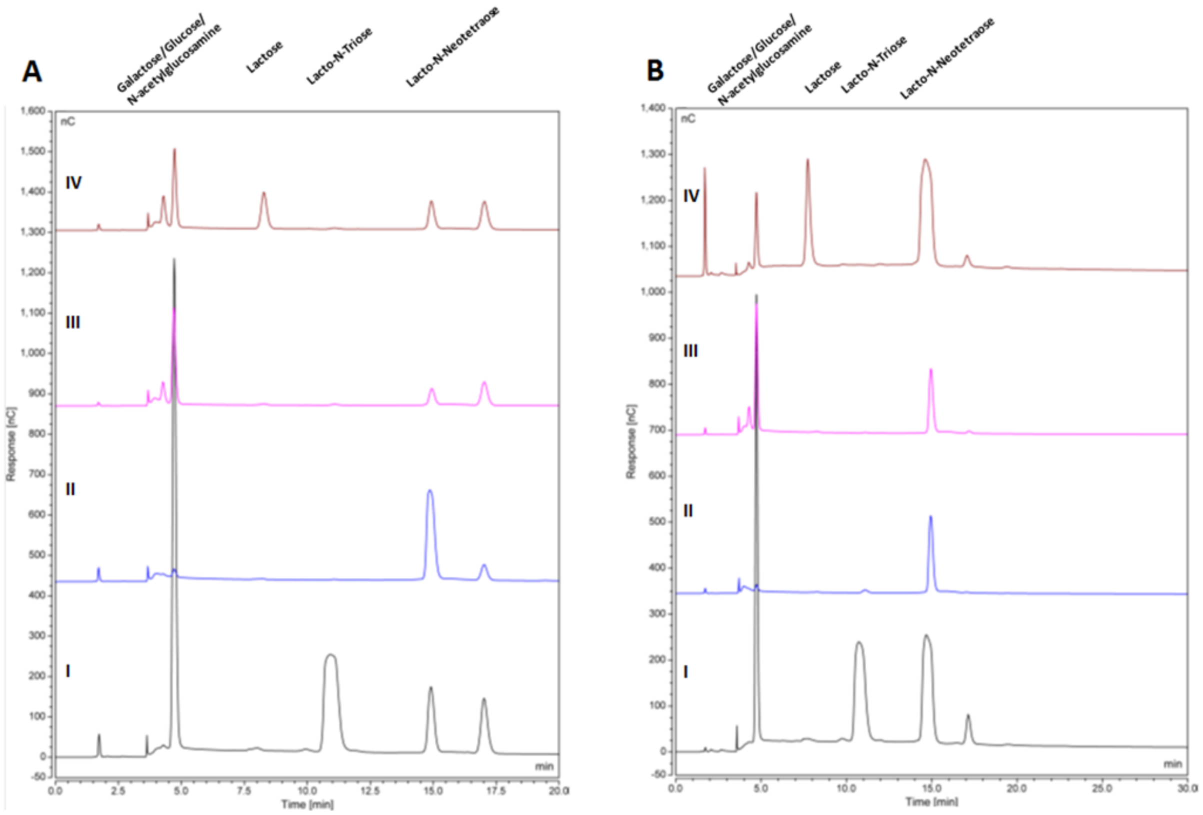

Supplementary Figure S2: (A) HPAEC chromatogram profiles of LNnT, when incubated in MOPS buffer (pH7) with: (I) LacZ2 alone, (II) NahA alone, (III) LacZ2 and NahA together, and (IV) LacZ2, followed by a denaturation step and the subsequent addition of NahA. **(B)** HPAEC chromatogram profiles of LNnT, when incubated in MOPS buffer (pH7) with: (I) LacZ6 alone, (II) NahA alone, (III) LacZ6 and NahA together, and (IV) LacZ6, followed by a denaturation step and the subsequent addition of NahA.

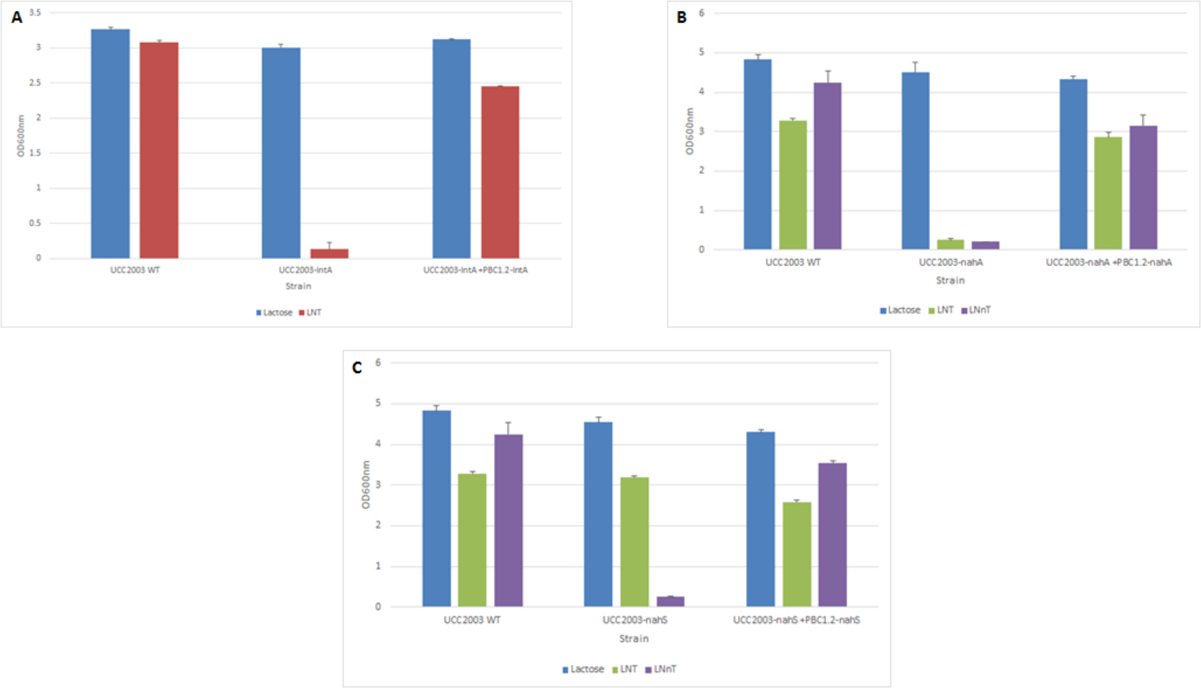

Supplementary Figure S3: (A) Final OD600 values (measured manually) after 24 hours of growth of wild type B. breve UCC2003, the insertion mutant *B. breve* UCC2003-lntA, and the complementation strain *B. breve* UCC2003-lntA+PBC1.2-lntA in modified MRS containing 1 % (wt/vol) lactose or 1 % (wt/vol) LNT as the sole carbon source. The results are the mean values obtained from two separate experiments*. Error bars represent the standard deviation. **(B)** Final OD600 values (measured manually) after 24 hours of growth of wild type B. breve UCC2003, the insertion mutant *B. breve* UCC2003-nahA, and the complementation strain *B. breve* UCC2003-nahA+PBC1.2-nahA in modified MRS containing 1 % (wt/vol) lactose or 1 % (wt/vol) LNT as the sole carbon source. The results are the mean values obtained from two separate experiments*. Error bars represent the standard deviation. **(C)** Final OD600 values (measured manually) after 24 hours of growth of wild type B. breve UCC2003, the insertion mutant *B. breve* UCC2003-nahS, and the complementation strain *B. breve* UCC2003-nahS+PBC1.2-nahS in modified MRS containing 1 % (wt/vol) lactose or 1 % (wt/vol) LNT as the sole carbon source. The results are the mean values obtained from two separate experiments*. Error bars represent the standard deviation.

*Experiments were carried out in duplicate due to a limited amount of carbohydrate material.

References:

1 Law, J. *et al.* A system to generate chromosomal mutations in Lactococcus lactis which allows fast analysis of targeted genes. *J Bacteriol* **177**, 7011-7018 (1995).

2 O'Connell Motherway, M., O'Driscoll, J., Fitzgerald, G. F. & Van Sinderen, D. Overcoming the restriction barrier to plasmid transformation and targeted mutagenesis in Bifidobacterium breve UCC2003. *Microb Biotechnol* **2**, 321-332, DOI:10.1111/j.1751-7915.2008.00071.x (2009).

3 de Ruyter, P. G., Kuipers, O. P. & de Vos, W. M. Controlled gene expression systems for Lactococcus lactis with the food-grade inducer nisin. *Applied and environmental microbiology* **62**, 3662-3667 (1996).

4 O'Connell Motherway, M., Kinsella, M., Fitzgerald, G. F. & van Sinderen, D. Transcriptional and functional characterization of genetic elements involved in galacto-oligosaccharide utilization by Bifidobacterium breve UCC2003. *Microb Biotechnol* **6**, 67-79, DOI:10.1111/1751-7915.12011 (2013).

5 Maze, A., O'Connell-Motherway, M., Fitzgerald, G. F., Deutscher, J. & van Sinderen, D. Identification and characterization of a fructose phosphotransferase system in Bifidobacterium breve UCC2003. *Applied and environmental microbiology* **73**, 545-553, DOI:10.1128/aem.01496-06 (2007).

6 Ruiz, L., Motherway, M. O., Lanigan, N. & van Sinderen, D. Transposon mutagenesis in Bifidobacterium breve: construction and characterization of a Tn5 transposon mutant library for Bifidobacterium breve UCC2003. *PLoS One* **8**, e64699, DOI:10.1371/journal.pone.0064699 (2013).

7 Egan, M. *et al.* Cross-feeding by Bifidobacterium breve UCC2003 during co-cultivation with Bifidobacterium bifidum PRL2010 in a mucin-based medium. *BMC microbiology* **14**, 282, DOI:10.1186/s12866-014-0282-7 (2014).

8 Álvarez-Martín, P., O'Connell-Motherway, M., van Sinderen, D. & Mayo, B. Functional analysis of the pBC1 replicon from Bifidobacterium catenulatum L48. *Applied microbiology and biotechnology* **76**, 1395-1402, DOI:10.1007/s00253-007-1115-5 (2007).

9 Mierau, I. & Kleerebezem, M. 10 years of the nisin-controlled gene expression system (NICE) in Lactococcus lactis. *Applied microbiology and biotechnology* **68**, 705-717, DOI:10.1007/s00253-005-0107-6 (2005).

10 McGrath, S., Fitzgerald, G. F. & van Sinderen, D. Improvement and optimization of two engineered phage resistance mechanisms in Lactococcus lactis. *Applied and environmental microbiology* **67**, 608-616, DOI:10.1128/aem.67.2.608-616.2001 (2001).
